# ExpaRNA-P: simultaneous exact pattern matching and folding of RNAs

**DOI:** 10.1186/s12859-014-0404-0

**Published:** 2014-12-31

**Authors:** Christina Otto, Mathias Möhl, Steffen Heyne, Mika Amit, Gad M Landau, Rolf Backofen, Sebastian Will

**Affiliations:** Bioinformatics, Institute of Computer Science, University of Freiburg, Freiburg, Germany; Bioinformatics, Department of Computer Science, University of Leipzig, Leipzig, Germany; Max Planck Institute of Immunobiology and Epigenetics, Stuebeweg 51, Freiburg, 79108 Germany; Department of Computer Science, University of Haifa, Mount Carmel, Haifa, Israel; Department of Computer Science and Engineering, NYU-Poly, Brooklyn, NY USA; Center for Biological Signaling Studies (BIOSS), University of Freiburg, Freiburg, Germany; Centre for Biological Systems Analysis (ZBSA), University of Freiburg, Freiburg, Germany; Center for non-coding RNA in Technology and Health, University of Copenhagen, Grønnegårdsvej 3, Frederiksberg C, DK-1870 Denmark

**Keywords:** RNA bioinformatics, Structure-based comparison of RNA, Sparsification

## Abstract

**Background:**

Identifying sequence-structure motifs common to two RNAs can speed up the comparison of structural RNAs substantially. The core algorithm of the existent approach ExpaRNA solves this problem for *a priori known* input structures. However, such structures are rarely known; moreover, predicting them computationally is no rescue, since single sequence structure prediction is highly unreliable.

**Results:**

The novel algorithm ExpaRNA-P computes exactly matching sequence-structure motifs in entire Boltzmann-distributed structure ensembles of two RNAs; thereby we match and fold RNAs simultaneously, analogous to the well-known “simultaneous alignment and folding” of RNAs. While this implies much higher flexibility compared to ExpaRNA, ExpaRNA-P has the same very low complexity (quadratic in time and space), which is enabled by its novel structure ensemble-based sparsification. Furthermore, we devise a generalized chaining algorithm to compute compatible subsets of ExpaRNA-P’s sequence-structure motifs. Resulting in the very fast RNA alignment approach ExpLoc-P, we utilize the best chain as anchor constraints for the sequence-structure alignment tool LocARNA. ExpLoc-P is benchmarked in several variants and versus state-of-the-art approaches. In particular, we formally introduce and evaluate strict and relaxed variants of the problem; the latter makes the approach sensitive to compensatory mutations. Across a benchmark set of typical non-coding RNAs, ExpLoc-P has similar accuracy to LocARNA but is four times faster (in both variants), while it achieves a speed-up over 30-fold for the longest benchmark sequences (≈400nt). Finally, different ExpLoc-P variants enable tailoring of the method to specific application scenarios. ExpaRNA-P and ExpLoc-P are distributed as part of the LocARNA package. The source code is freely available at http://www.bioinf.uni-freiburg.de/Software/ExpaRNA-P.

**Conclusions:**

ExpaRNA-P’s novel ensemble-based sparsification reduces its complexity to quadratic time and space. Thereby, ExpaRNA-P significantly speeds up sequence-structure alignment while maintaining the alignment quality. Different ExpaRNA-P variants support a wide range of applications.

**Electronic supplementary material:**

The online version of this article (doi:10.1186/s12859-014-0404-0) contains supplementary material, which is available to authorized users.

## Background

Genome-wide high-throughput transcriptomics has revealed evidence for massive transcription of eukaryotic genomes, vastly exceeding translation to proteins [1-3]. Ultimately, the ENCODE project [4] has established pervasive transcription of most of both strands of the human genome. Remarkably, while only a minor fraction of the transcripts codes for proteins, the majority of the non-coding RNAs (ncRNAs) are associated with function [5]. Nevertheless, the functional annotation is lagging behind strongly: reliable automated annotation pipelines exist only for subclasses of ncRNAs like tRNAs, microRNAs, or snoRNAs [6].

Recent computational screens, e.g. [7], reveal stable, conserved structures in a large part of ncRNAs, again pointing to function. The *de novo* RNA-gene finders qrna [8], MSARi [9], EvoFold [10], and RNAz [11] identify conservation of stable RNA structures in whole genome alignments; this can be boosted by structure-based realignment (REAPR [12]). Identifying RNAs with similar sequence and common secondary structure advances further towards the automatic annotation of non-coding RNAs. At genomic scale, clustering approaches like [13-15] identify remote members of RNA-*families* as defined in the Rfam database [16], and determine new *classes* of structurally similar – hence, likely functionally related – ncRNAs. Thus, all such analysis of RNAs relies on comparing RNAs.

### Simultaneous alignment and folding (SA&F)

Aligning RNAs and, simultaneously, inferring their common structure is considered the gold standard for comparing RNAs. [17] solves this problem in *O*(*n*^6^) time and *O*(*n*^4^) space (for RNAs of length *n*). In practice, e.g. for searching remote members of RNA-families, this complexity is strongly limiting. Even worse, identifying novel RNA-classes in the plethora of newly discovered RNA-transcripts (by all-against-all pairwise comparisons) is simply not feasible by Sankoff’s SA&F method.

Many Sankoff-implementations [18-24] reduce the high computational demands by sequence-based heuristics. A prominent line restricts the search space based on alignment probabilities that consider only sequence information. This idea was introduced by [20], and later refined by [22] and [24].

PMcomp [25] introduced an orthogonal idea to gain speed up over Sankoff’s algorithm. Applying a lightweight energy model, which assigns energies to single base pairs, enables to lower computational costs significantly. For – at the same time – high accuracy, it scores structural matches by ensemble base pair probabilities, precomputed in a full-featured energy model [26]. LocARNA [13] implements the lightweight energy model of PMcomp, but gains further speed-up by introducing the structure-based heuristic *ensemble-based sparsification*. Employing the structural sparsity of RNA structure ensembles, LocARNA’s complexity is improved to only *O*(*n*^4^) time and *O*(*n*^2^) space.

Subsequently, other Sankoff-like methods [27-29] apply similar ensemble-based sparsifications in lightweight models. RAF [29] additionally inherits the sequence-based speed up of [24].

In [30], we have proposed the lightweight SA&F strategy ExpLoc; it cuts down the computational demands significantly beyond LocARNA’s sparsification, but unlike other heuristic improvements such as [29], ExpLoc does not restrict the search space based on structure-ignorant sequence alignments. ExpLoc computes exactly conserved elements in pairs of *fixed* RNA secondary structures, based on an algorithm with quadratic time and space complexity [31]; subsequently, these elements provide anchors for a LocARNA alignment. In hindsight, this strategy suffers from similar problems as the first generation of RNA alignment methods [32,33]: relying on a single predicted input structure for each sequence, this strategy fails frequently and causes severe misalignments, since predicting minimum free energy structures from single sequences is highly unreliable.

### Simultaneous matching and folding (SM&F)

Here, we present a novel algorithm that enables an ExpLoc-like speed-up while resolving its fundamental problem (of relying on fixed structures) by performing exact matching and RNA folding simultaneously. Studying exact matching in non-fixed structures with a very different focus, we have discussed heavy path decomposition for related problems [34]; furthermore, we have presented preliminary work on ensemble-based exact matching in [35]. The novel algorithm ExpaRNA-P computes exactly sequence-structure-conserved elements that form highly probable local substructures in the RNA structure ensembles of both input RNAs.

Analogous to Sankoff’s SA&F idea, the novel strategy performs “simultaneous matching and folding” (SM&F) of RNA sequences. Thereby, it liberates exact pattern matching from its restriction to *a priori* fixed structure [31]. We point out that a straight-forward extension of the fixed input structure matching to SM&F, would require at least *O*(*n*^4^) time and *O*(*n*^2^) space, which is still as high as the complexity of LocARNA. However, to speed up RNA comparison significantly, reducing this complexity is fundamental.

### Sparsification of SM&F

Thus, our main technical contribution is to solve SM&F in quadratic time and space — as efficiently as plain sequence alignment. This is enabled by a novel sparsification technique that substantially goes beyond prior approaches. Utilizing novel ensemble properties of the sequences, we identify sparse regions of each matrix such that, in total across all matrices, only quadratically many *matrix entries* have to be computed; each of them calculated in constant time. In contrast, LocARNA reduces only the number of computed *DP-matrices*, but requires quadratic time for each of them. This novel sparsification is based on limiting the joint probability of a sequence position or a base pair occurring as parts of particular loops in the ensembles of the single RNAs.

Notably, other sparsification approaches [36-39] apply a different (not *ensemble-based*) form of sparsification. Generally, these methods rule out subsolutions, which are computed by the DP, that can not occur in the optimal solution. This allows deriving a provably optimal solution while reducing the number of required case distinctions. In contrast, the idea of our sparsification is to remove subsolutions that are unlikely in the solution ensemble. Consequently, ensemble-based sparsification does not only allow much stronger savings, but moreover is applicable even for computing partition functions of RNA alignments; this is realized in LocARNA-P [40], which computes RNA alignment reliabilities from SA&F partition functions.

### Overview of results

**Figure 1:**
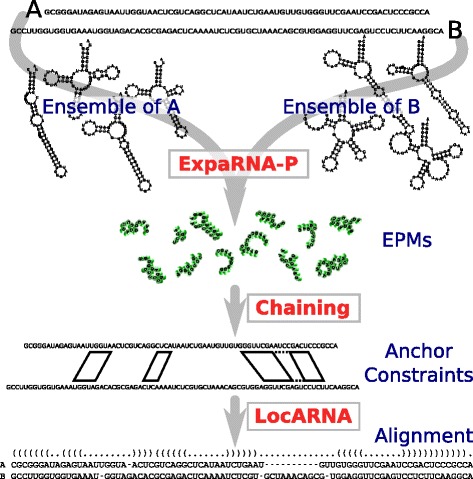
**The** **ExpLoc-P** **pipeline.** Using EPMs as anchor constraints to speed up RNA structure alignments.

To evaluate the practical benefits of our algorithmic innovations, we construct the pipeline ExpLoc-P for SA&F (in the spirit of ExpLoc), which we sketch in Figure 1. In its first stage, it enumerates suboptimal exact matchings of local sequence-structure patterns due to the introduced algorithm ExpaRNA-P. In the second stage, the suboptimal matchings are chained to select an optimal subset of compatible matchings that can simultaneously occur in an alignment of RNAs. Finally, these matchings are heuristically utilized as anchor constraints in the subsequent LocARNA alignment.

First, we study important design choices in the ExpLoc-P pipeline, which provides insights into practical implications of the developed concepts; in particular, we compare strict and relaxed matching in ExpaRNA-P. The latter allows mismatches at structural positions, which improves the coverage of low identity sequences. In extensive benchmarks, ExpLoc-P produces high-quality alignments. At the same time, due to its heuristic use of ExpaRNA-P anchors, it achieves a considerable speed-up (about four-fold) over the benchmark set of typical RNAs (BRAliBase 2.1). For long sequences (≈400 nt) of the benchmark set, the speed-up is more than 30-fold.

## Methods

### Preliminaries

An *RNA sequence A* is a string over the alphabet {*A*,*C*,*G*,*U*}. *A*_*i*_ denotes the *base at the i-th position of A*; *A*_*i*..*j*_, the *substring of A from position i to j*, which is called *subsequence* in this context; and |*A*|, the *length of A*. A *structure* of A is a set *S* of base pairs (*i*,*j*) such that 1≤*i*<*j*≤|*A*|, where *A*_*i*_ and *A*_*j*_ are complementary (A-U, C-G, or G-U.) Furthermore, structures are *non-crossing*: in a structure *S*, each sequence position is involved in at most one base pair, i.e. for all (*i*,*j*),(*i*^′^,*j*^′^)∈*S*: (*i*=*i*^′^⇔*j*=*j*^′^) and *i*≠*j*^′^, and base pairs do not *cross*, i.e. there are no base pairs (*i*,*j*),(*i*^′^,*j*^′^)∈*S* s.t. *i*<*i*^′^<*j*<*j*^′^. The *span* of a base pair (*i*,*j*) is *j*−*i*.

Let *S* be a structure of sequence *A*. We define the *pseudo-base pairψ*_*A*_:=(0,|*A*|+1). The *parent of position k in S* is the base pair (*i*,*j*)∈*S*∪*ψ*_*A*_ with *i*<*k*<*j* such that there does not exist any (*i*^′^,*j*^′^)∈*S* with *i*<*i*^′^<*k*<*j*^′^<*j*. Analogously, the *parent of a base pair (i,j)* is the parent of *i* (which is the parent of *j* at the same time). Note that parents are unique, since non-crossing structures correspond to trees.

Furthermore, we define loop_*S*_(*i*,*j*) as the set of positions of *A* and base pairs in *S*, whose parent in *S* is (*i*,*j*); note that loop_*S*_(*i*,*j*) is empty, if (*i*,*j*)∉*S*. Intuitively, if a base or base pair has the parent (*i*,*j*) in *S*, it belongs to the loop closed by (*i*,*j*) in *S*.

For a sequence *A*, let Pr[*S*|*A*] denote the probability of the structure *S* in the Boltzmann ensemble of *A* [26]. Pr[(*i*,*j*)|*A*] denotes the *base pair probability of (i,j)*, which is defined as $\sum _{S\ni (i,j)} \Pr \left [\vphantom {\Pr {\left [(i,j)|A\right ]}}S|A\right ]$. Thus, Pr[(*i*,*j*)|*A*] is the probability that a random structure *S*, drawn from the Boltzmann ensemble of *A*, contains the base pair (*i*,*j*).

### Pattern matchings in RNA structure ensembles

**Figure 2:**
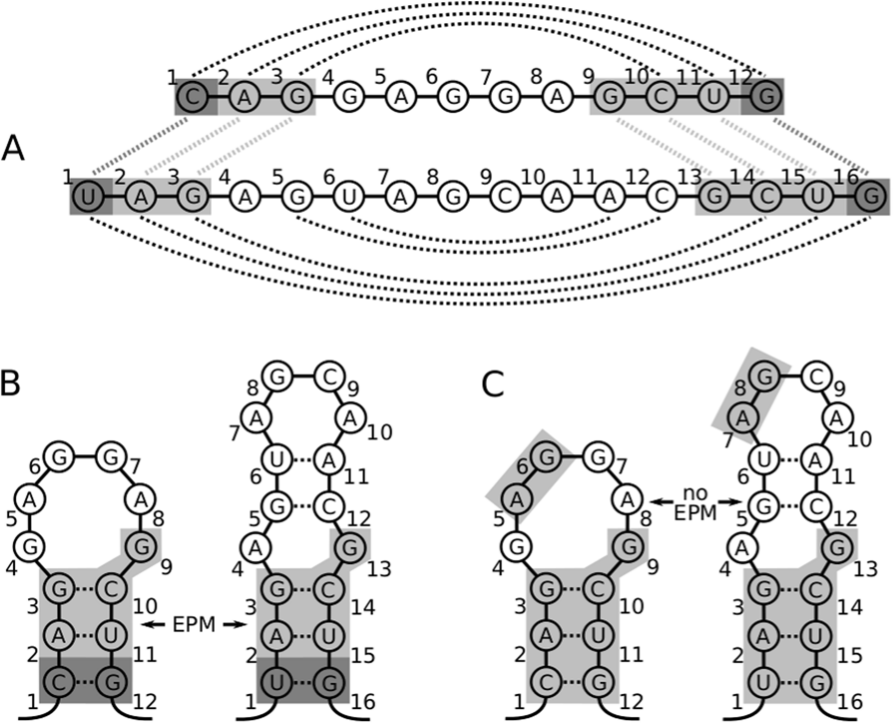
**Visualization of the pattern matching definition.** Two different illustrations of the notion pattern matching are shown in **(A)** and **(B)**. For the light gray pattern matching, we have $\mathcal {M} = \{2\!\sim \!2,3\!\sim \!3,9\!\sim \!13,10\!\sim \!14,11\!\sim \!15\}$ and $\mathcal {S} = \{(2,11\!\sim \!2,15), (3,10\!\sim \!3,14)\}$. Note that the two separated regions in both sequences are connected through base pairs. Furthermore, the set of structure matches is $\mathcal {M}\vert _{\mathcal {S}}=\{2\!\sim \!2,3\!\sim \!3,10\!\sim \!14,11\!\sim \!15\}$ and the set of sequence matches is $\mathcal {M} \setminus \mathcal {M}\vert _{\mathcal {S}} =\{9\!\sim \!13\}$. The pattern matching can be extended by the base pair match shown in dark gray, i.e., $\mathcal {M}' = \mathcal {M} \cup \{1\!\sim \!1,12\!\sim \!16\}$ and $\mathcal {S}' = \mathcal {S} \cup \{(1,12\!\sim \!1,16)\}$. $(\mathcal {M},\mathcal {S})$, the EPM shown in light gray, is a strict EPM, whereas $(\mathcal {M}',\mathcal {S}')$, the EPM extended by the dark gray part, is a relaxed EPM as mismatches in structure matches occur. **(C)** shows an example of an invalid matching. Separately, both the small and the big matched gray parts are valid EPMs, but together they do not form a valid EPM as the two individual parts are not connected.

ExpaRNA-P identifies sequence-structure patterns that are shared by two input RNA sequences. We provide a general description of pattern matchings in RNA sequences and specialize to two different variants (for examples, see Figure 2). We fix sequences A and B with lengths |*A*|=*n* and |*B*|=*m*; for stating computational complexities, we assume *m*≤*n*. The sets of possible base pairs of respective sequences *A* and *B* are denoted by *P* and *Q*.

#### **Definition1** (connected, Pattern Matching).

We denote the *match of positions i and k* by *i* ∼ *k* and the *base pair match of base pairs (i,j) and (k,l)* by *ij* ∼ *kl*. We consider pairs  of arbitrary sets $\mathcal {M}\subseteq \{i\!\sim \!k \mid i\in [\!1..n],k\in [\!1..m]\!\}$ and $\mathcal {S}\subseteq \{ij\!\sim \!kl \mid (i,j)\in [\!1..n]^{2}, i<j, (k,l)\in [\!1..m]^{2},k<l\}$. $\mathcal {P}=(\mathcal {M},\mathcal {S})$ is *connected*, iff the graph $\mathcal {G}_{\mathcal {P}}=(\mathcal {M},\mathcal {E})$, where $\mathcal {E}=\{(i\!\sim \!k,j\!\sim \!l) \mid (\,j=i+1 \text {~and~} l=k+1) \text {~or~} ij\!\sim \!kl\in \mathcal {S}\}$, is (weakly) connected.

 is called *Pattern Matching* iff

is a matching,
  i.e. *i*=*j*⇔*k*=*l* for all $i\!\sim \!k, j\!\sim \!l\in \mathcal {M}$
is non-crossing,
  i.e. *i*<*j*⇒*k*<*l* for all $i\!\sim \!k,j\!\sim \!l\in \mathcal {M}$
‘contains’ ,
  i.e. $ij\!\sim \!kl\in \mathcal {S}\Rightarrow \{i\!\sim \!k,j\!\sim \!l\}\subseteq \mathcal {M}$
- the structure $\{(i,j) \mid ij\!\sim \!kl\in {\mathcal S}\}$ is non-crossing
  (consequently, together with the previous condition, $\{(k,l) \mid ij\!\sim \!kl\in \mathcal {S}\}$ is non-crossing as well).
- $(\mathcal {M},\mathcal {S})$ is connected.

A position *i* is *matched by**(in sequence A)* iff there is a position *k*, s.t. $i\!\sim \!k\in \mathcal {M}.$ This is symmetrically defined for positions *j* and sequence *B*.

We are going to define strict and relaxed exact pattern matchings (cf. Figure 2AB). In the former, all matched nucleotides have to be identical. The latter relaxes this by allowing mismatched nucleotides at matched base pairs (taking compensatory mutations into account).

For this purpose, we distinguish two kinds of matches in a pattern matching $({\mathcal M},{\mathcal S})$: define the set of structure matches as $\mathcal {M}\vert _{\mathcal {S}}:=\{i\!\sim \!k,j\!\sim \!l \mid ij\!\sim \!kl\in \mathcal {S}\}$; the set of sequence matches is

$$\mathcal{M} \setminus \mathcal{M}\vert_{\mathcal{S}} = \{i\!\sim\!k \in \mathcal{M} \mid i\!\sim\!k \notin \mathcal{M}\vert_{\mathcal{S}}\}, $$

i.e. all matches that are not structural matches.

#### **Definition2** (Strict EPM).

A *strict Exact Pattern Matching (strict EPM)* is a pattern matching (Def. 1) with the additional property:

$$\text{for all}~ i\!\sim\!k \in \mathcal{M}: A_{i}=B_{k}. $$

#### **Definition3** (Relaxed EPM).

A *relaxed Exact Pattern Matching (relaxed EPM)* is a pattern matching with the additional property:

$$\text{for all}~ i\!\sim\!k \in \mathcal{M} \setminus \mathcal{M}\vert_{\mathcal{S}} : A_{i}=B_{k}. $$

We introduce the term EPM to refer to strict EPMs and relaxed EPMs generically. By Definition 1, a pattern matching, and therefore an EPM, does not necessarily match positions of contiguous subsequences, but it is required that the matched sequence-structure motifs are *structure-local* [41,42] in each sequence. For example, in Figure 2B, the sets of gray sequence positions in each RNA are *structure-local*, because these positions are (graph-theoretically) connected via edges formed by backbone or base pair bonds; in contrast gray motifs in Figure 2C are not structure-local, because they consist of two separated connected components.

To characterize good EPMs, we define the *score of an EPM *$(\mathcal {M},\mathcal {S})$ by summing up single score contributions of base and base pair matches:

(1) $$ \text{score}(\mathcal{M},\mathcal{S})=\!\!\sum_{i\sim k\in \mathcal{M} \setminus \mathcal{M}\vert_{\mathcal{S}}}\!\!\sigma(i,k)\ + \sum_{ij\sim kl \in \mathcal{S}}\!\!\tau(i,j,k,l),  $$

where *σ* and *τ* are scoring functions with properties *σ*(*i*,*k*)>0 if *A*_*i*_=*B*_*k*_ and *τ*(*i*,*j*,*k*,*l*)>0 if *A*_*i*_=*B*_*k*_ and *A*_*j*_=*B*_*l*_. In our studies, we set *σ*(*i*,*k*) to 1 if *A*_*i*_=*B*_*k*_ (otherwise, −*∞*); furthermore, *τ* is parameterized by

(2) $$ \begin{aligned} \tau(i,j,k,l)&= \alpha_{1} \left(\text{c\_seq}(i,k) + \text{c\_seq}(j,l)\right) \\ &\hspace*{12pt} + \alpha_{2} \text{c\_str}(i,j,k,l) \\ &\hspace*{12pt}+ \alpha_{3} \text{c\_sta}(i,j,k,l) \\ \text{c\_seq}(i,k)&= \begin{cases} 1 & \text{if}~A_{i} = B_{k} \\ \text{str\_mm} & A_{i} \neq B_{k} \end{cases}\\ \text{c\_str}(i,j,k,l)&= \Pr\left[{(i,j)|A}\right]+\Pr\left[\vphantom{\Pr{\left[(i,j)|A\right]}}{(k,l)|B}\right]\\ \text{c\_sta}(i,j,k,l)&= \Pr\left[{(i,j) \land (i+1,j-1)|A}\right]\\ &\hspace*{12pt}+ \Pr\left[\vphantom{{(i,j) \land (i+1,j-1)|A}}{(k,l) \land (k+1,l-1)|B}\right] \end{aligned}  $$

The parameters *α*_1_, *α*_2_, and *α*_3_ weight respective contributions of sequence matches, structure matches, and stacking. The stacking contribution *c_sta* rewards stacked base pairs. Each mismatch at the left or right end of a base pair match is penalized by *str_mm*; for scoring strict EPMs, we set this penalty to −*∞*, which forbids all kinds of mismatches. In analogy to the notation Pr[(*i*,*j*)|*A*], Pr[(*i*,*j*)∧(*i*+1,*j*−1)|*A*] denotes the joint probability of the stacked base pairs (*i*,*j*) and (*i*+1,*j*−1). Such probabilities are computed in slight extension of McCaskill’s algorithm [43].

As in the case of RNA structures (of some sequence *A*), one can define parent relations in EPMs of sequences *A* and *B*. In analogy, we define the *pseudo-base pair match* to match the two pseudo base pairs, i.e. *ψ*:=*ψ*_*A*_ ∼ *ψ*_*B*_. In the following, we consider the base pair matches *i*^′^*j*^′^ ∼ *k*^′^*l*^′^ to be order by their spans *j*^′^−*i*^′^ (or *k*^′^−*l*^′^; the choice is arbitrary, since we consider only non-crossing structure.) According to this partial order, we define $\text {parent}_{\mathcal {S}}(i\!\sim \!k)$ as the smallest $i'j'\!\sim \!k'l'\in \mathcal {S}\cup \{\psi \}$ that satisfies *i*^′^≤*i*≤*j*^′^; $\text {parent}_{\mathcal {S}}(ij\!\sim \!kl)$ denotes the smallest base pair match that satisfies *i*^′^<*i*<*j*<*j*^′^.

We define additional joint probabilities to characterize the “interesting” EPMs.

#### **Definition4** (Joint probabilities).

We define joint occurrence probabilities of elements in loops of structures in the Boltzmann ensemble of *X*, where *X* denotes either *A* or *B*.

- Pr [*k* ∈ *loop*(*i*,*j*)|*X*] denotes for *i*<*k*<*j* the joint probability that a structure of *X* contains the base pair (*i*,*j*)*and* the unpaired base *k* such that (*i*,*j*) is the parent of *k*.
- Pr [(*i*^′^,*j*^′^) ∈ *loop*(*i*,*j*)|*X*] denotes for *i*<*i*^′^<*j*^′^<*j* the joint probability that a structure of *X* contains the base pairs (*i*,*j*)*and* (*i*^′^,*j*^′^) such that (*i*,*j*) is the parent of (*i*^′^,*j*^′^).

For catchy notation, the expressions loop(*i*,*j*) in Def. 4 resemble loop_*S*_(*i*,*j*) – notationally omitting the structures *S* in the Boltzmann ensemble of *A* (analogously, *B*).

We introduce an efficient algorithm to compute these probabilities in Section ‘Precomputation: joint loop probabilities’. Since we want to match only structures that have high probability in the Boltzmann ensembles of the given sequences – as computed by McCaskill’s algorithm [26] – we define the notion of significant EPMs. This constraint is crucial for both the quality of the results and the complexity of the algorithm. To define significance, we furthermore introduce three thresholds *θ*_1_,*θ*_2_ and *θ*_3_. We limit the probability of all matched base pairs by *θ*_1_; furthermore, the joint probabilities of matched unpaired bases and base pairs, occurring as part of their enclosing loop, by *θ*_2_ and *θ*_3_, respectively.

#### **Definition5** (Significant EPMs).

Given thresholds *θ*_1_,*θ*_2_, and *θ*_3_, an EPM is *significant* iff

- for all $ij\sim kl \in \mathcal {S}$:
  Pr[(*i*,*j*)|*A*]≥*θ*_1_ and Pr[(*k*,*l*)|*B*]≥*θ*_1_
- for all $i \sim k \in \mathcal {M} \setminus \mathcal {M}\vert _{\mathcal {S}}$:
  Pr[*i*∈loop(*i*^′^,*j*^′^)|*A*]≥*θ*_2_
  and Pr[*k*∈loop(*k*^′^,*l*^′^)|*B*]≥*θ*_2_,
  where $i'j' \sim k'l'=\text {parent}_{\mathcal {S}}(i \sim k) \neq \psi $
- for all $ij \sim kl \in \mathcal {S}$:
  Pr[(*i*,*j*)∈loop(*i*^′^,*j*^′^)|*A*]≥*θ*_3_
  and Pr[(*k*,*l*)∈loop(*k*^′^,*l*^′^)|*B*]≥*θ*_3_,
  where $i'j' \sim k'l'=\text {parent}_{\mathcal {S}}(ij \sim kl) \neq \psi $

We reduce the return set of our algorithm further by reporting only EPMs that are not included in better (reported) EPMs and that do not include better EPMs. The second condition is relevant only for relaxed EPMs, since this cannot occur for strict EPMs. In the case of strict EPMs, those EPMs are simply *maximal* w.r.t. the following inclusion order $\sqsubseteq $ of pattern matchings. Hence, we call them *maximal strict EPMs*.

#### **Definition6** (Inclusion Order on EPMs).

Let $\mathcal {P} = (\mathcal {M},\mathcal {S})$ and $\mathcal {P}' = \left (\mathcal {M}'\hspace *{.01pt},\mathcal {S}'\right)$ be EPMs.  is included in $\mathcal {P}'$, written $\mathcal {P}\sqsubseteq \mathcal {P}'$ iff

- $\mathcal {M} \subseteq \mathcal {M}'$
- for all $i\!\sim \!k\in \mathcal {M}$:
  $\text {parent}_{\mathcal {S}}(i\!\sim \!k)=\text {parent}_{\mathcal {S}'}(i\!\sim \!k)$

**Figure 3:**
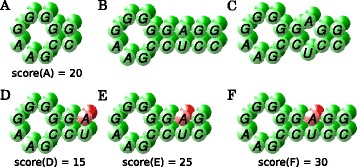
**Visualization of maximal EPMs.** Matches of green bases refer to exact matches and red ones to inexact (structure) matches. **(A-C)** EPM A is not maximal since there exists a larger (strict) EPM **(B or C)**. EPMs **B** and **C** can be maximal simultaneously since in each case some base matches have different parents. **(A and D-F)** EPM D is generated from A by appending an inexact structure match and has a lower score than A. Further extending the EPM leads to higher scores again **(E and F)**. **D** is not maximal since A has the same parents and a higher score. **A** is not maximal because there exist (relaxed) EPMs **E** and **F** with the same parents and higher scores. Among **A**, **D**, **E**, and **F**, only **F** is maximal.

Notably, in the inclusion order of Def. 6, EPMs with different structures are not comparable. Consequently, two EPMs that match the same positions can be both maximal, if they match different structure. This is illustrated in Figure 3 (A-C).

In the case of strict EPMs, the highest scoring EPMs are always maximal EPMs w.r.t. the inclusion order, which allows us to select the “interesting” EPMs by this simple property. However, the same does not hold for relaxed EPMs: for example, typically the score of a relaxed EPM decreases if it is extended by a structure match with mismatching nucleotides; still, further extensions can increase the total score again. These dependencies are illustrated in Figure 3 (A and D-F).

Consequently, since we want to keep the highest scoring EPMs in the case of relaxed EPMs as well, we define a score-extended partial order.

#### **Definition7** (Score Inclusion Order).

Let $\mathcal {P} = (\mathcal {M},\mathcal {S})$ and $\mathcal {P}' = (\mathcal {M}',\mathcal {S}')$ be EPMs.  is smaller than $\mathcal {P}'$ in the score inclusion order, iff $\text {score}(\mathcal {P}) < \text {score}(\mathcal {P}')$ and ($\mathcal {P} \sqsubseteq \mathcal {P}'$ or $\mathcal {P}' \sqsubseteq \mathcal {P}$).

We call a relaxed EPM *maximal*, iff it is maximal w.r.t. this order among all relaxed EPMs. In other words, a relaxed EPM is maximal if and only if there is no second relaxed EPM with a higher score that is, by inclusion order, (a) smaller or (b) larger in the relaxed EPM (see Figure 3 (A and D-F)). Note that different patterns with the same score are not comparable so that they cannot rule out each other.

Both maximality definitions are canonically raised to *maximal significant* strict EPMs and relaxed EPMs.

### Precomputation: joint loop probabilities

Fundamentally, our novel sparsification technique relies on the joint probabilities of Def. 4. For sequences *X*∈{*A*,*B*}, one efficiently computes base pair probabilities Pr[(*i*,*j*)|*X*] by McCaskill’s algorithm [26]. In this work, we extend this algorithm to compute the probabilities Pr[*k* ∈ loop(*i*,*j*)|*X*] and Pr[(*i*^′^,*j*^′^) ∈ loop(*i*,*j*)|*X*] for *X*∈{*A*,*B*}. For this purpose, we introduce – on top of the McCaskill matrices – the auxiliary matrix $Q_{\textit {ij}}^{m2}$, which represents parts of a multiloop with at least two outermost base pairs. This enables computing the additional joint probabilities efficiently in the complexity bounds of the McCaskill algorithm (Additional file 1).

Importantly, all these probabilities are efficiently precomputed independently for each sequence. Hence, e.g. in clustering scenarios, where all pairs from a set of sequences need to be matched, this preprocessing needs to be done only once for each sequence and not for all quadratically many pairs.

### ExpaRNA-P: Optimizing over significant EPMs

**Figure 4:**
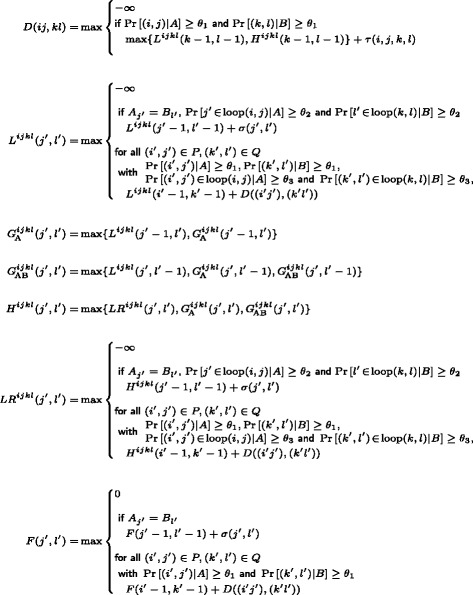
**Recursion equations.** Recursions for computing the significant strict EPMs and relaxed EPMs, respectively. These equations are visualized in Figure 5.

**Figure 5:**
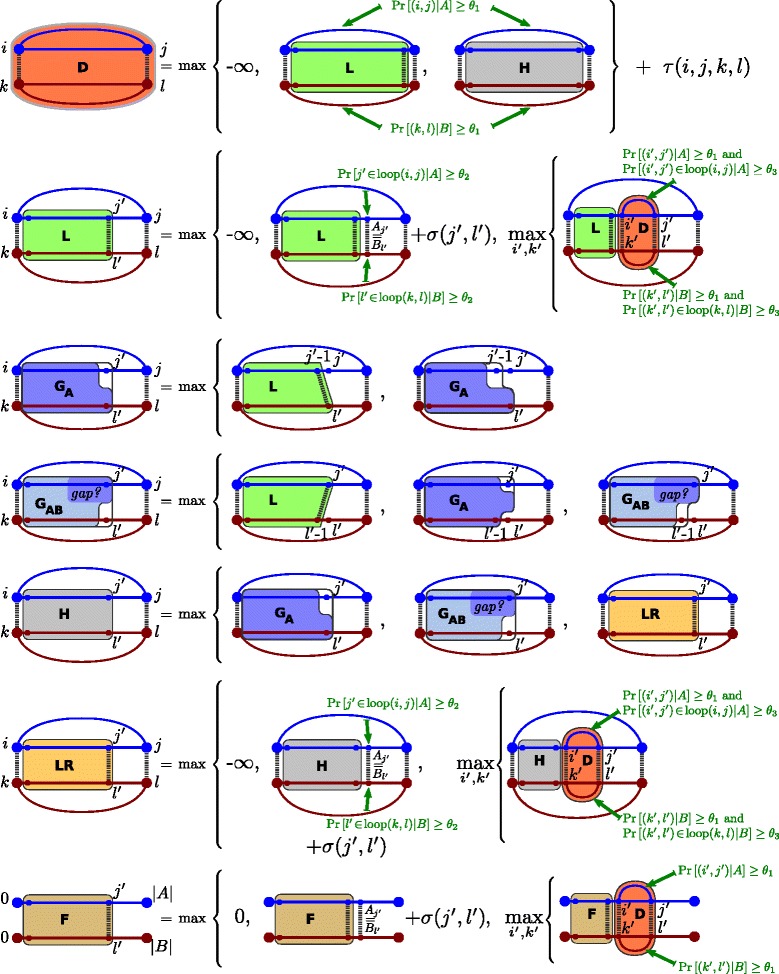
**Recursion visualization.** Visualization of the recursions to compute the matrix entries $L^{ijkl}(j',l'), G_{\!\text {A}}^{ijkl}(j',l'), G_{\!\text {AB}}^{ijkl}(j',l'), LR^{ijkl}(j',l'), D(ij,kl), F(j',l')$ and the auxiliary matrix *H* ^*ijkl*^(*j* ^′^,*l* ^′^).

Figure 4 provides formal recursion equations of the dynamic programming EPM optimization algorithm; the same recursions are presented graphically in Figure 5.

Fundamental to our approach, all matrices and evaluations in the recursions are sparse, i.e. only entries and cases are considered where the probabilities of elements pass the respective probability thresholds (cf. Def. 5). Corresponding constraints are given in the recursion equations – this is also illustrated in Figure 5, using arrows. Otherwise, we can largely postpone this aspect until Section ‘ExpaRNA-P: Sparsification’.

The matrix entries *D*(*ij*,*kl*) score the best EPM enclosed by each base pair match *ij* ∼ *kl*, i.e. *D*(*ij*,*kl*) denotes the best score of a significant EPM $(\mathcal {M},\mathcal {S})$ of *A*_*i*..*j*_ and *B*_*k*..*l*_ with $ij\!\sim \!kl\in \mathcal {S}$.

Inside of the base pair match *ij* ∼ *kl*, we determine the (score of the) best $(\mathcal {M},\mathcal {S})$ that is either a significant EPM itself *or* forms a (connected) significant EPM only together with the closing base pair match *ij* ∼ *kl*. The first case is covered by the single matrix *L*, whereas the latter case requires three matrices *G*_A_, *G*_AB_, and *LR*. By and large, for deriving one *D*-entry one starts matching from the left using *L*. Potentially, one introduces a gap using matrices *G*_A_ and *G*_AB_ and continues using matrix *LR* to match the part that is only connected to the right end of *ij* ∼ *kl*.

In more detail, first we determine the best score of a significant EPM $\mathcal {P}=(\mathcal {M},\mathcal {S})$ that is connected to the left end *i* ∼ *k* of the base pair match, i.e.  is empty or contains *i*+1 ∼ *k*+1. Concretely, *L*^*ijkl*^(*j*^′^,*l*^′^) is such a score, where $\mathcal {M}\subseteq [i+1..j']\times [\!k+1..l']$ and $j'\!\sim \!l'\in \mathcal {M}$. To introduce a gap, the latter condition is changed for *G*_A_ and *G*_AB_. In the case of $G_{\!\text {A}}^{ijkl}(j',l'), \mathcal {M}$ does not match *j*^′^ but matches *l*^′^; for $G_{\!\text {AB}}^{ijkl}(j',l'), \mathcal {M}$ does not match *l*^′^ and potentially does not match *j*^′^. Finally, *LR*^*ijkl*^(*j*^′^,*l*^′^) is the best sum of scores of two significant EPMs $\mathcal {P}_{1}=(\mathcal {M}_{1},\mathcal {S}_{1})$ and $\mathcal {P}_{2}=(\mathcal {M}_{2},\mathcal {S}_{2})$ where the first is connected to the left base pair match end *i* ∼ *k* and the second contains *j*^′^ ∼ *l*^′^. Intuitively, the two EPMs are separated by a gap; formally: (for all $i_{1}\!\sim \!k_{1}\in \mathcal {M}_{1}$ and $i_{2}\!\sim \!k_{2}\in \mathcal {M}_{2}, i_{1}<i_{2}-1$ and *k*_1_<*k*_2_) or (for all $i_{1}\!\sim \!k_{1}\in \mathcal {M}_{1}$ and $i_{2}\!\sim \!k_{2}\in \mathcal {M}_{2}, i_{1}<i_{2}$ and *k*_1_<*k*_2_−1).

Our recursion equations (Figure 4 and Figure 5) show the precise case distinctions and dependencies. In *L*, we check whether there is a sequence match (second case) or a structure match (third case); otherwise, we assign −*∞* (first case). *LR* is analogous to *L*, only allowing to close a gap left of the structure or sequence match. For this purpose, we introduce an auxiliary matrix *H*, which does not need to be stored. The gap itself, computed in *G*_A_ and *G*_AB_, allows skipping an arbitrary number of positions in both sequences. The recursion structure ensures that such a gap is introduced at most once per loop match and sequence. To avoid ambiguity, the recursion enforces to first skip positions in *A* (using *G*_A_) and after that positions in *B* (using *G*_AB_); furthermore we enforce a gap in the matchings computed via *LR* by its initialization.

We compute entries of *D* in increasing order with respect to their size so that when computing some *D*(*ij*,*kl*), any *D*(*i*^′^*j*^′^,*k*^′^*l*^′^) with *i*<*i*^′^<*j*^′^<*j* and *k*<*k*^′^<*l*^′^<*l* is already computed.

Since EPMs are not necessarily closed by a base pair match (like the EPMs of *D*), we finally compute the matrix *F*. The entries *F*(*j*^′^,*l*^′^), for 0≤*j*^′^≤*n* and 0≤*l*^′^≤*m*, denote the maximum score of a significant EPM of ${A}_{1..j^{\prime }}\phantom {\dot {i}\!}$ and ${B}_{1..l^{\prime }}\phantom {\dot {i}\!}$, which ends at (*j*^′^,*l*^′^), i.e. with $j'\!\sim \!l'\in \mathcal {M}$. The recursion for *F* is almost identical to the recursion for *L*, except for the first case, which is 0 instead of −*∞*, since the EPMs in *F* can start at any point (similar to local sequence alignments). Also, since the matched base pairs in EPMs of *F* are external (i.e. they are not enclosed by some other base pair of the EPM), we do not perform checks for the second and third condition of significant EPMs (Def. 5).

**Matrix initialization** Matrix entries corresponding to matches of empty subsequences are initialized. Here, we take special care to disallow such matches for certain matrices (by assigning −*∞*).

- $L^{ijkl}(i,k)=G_{\!\text {A}}^{ijkl}(i,k)=G_{\!\text {AB}}^{ijkl}(i,k)=0$ and *LR*^*ijkl*^(*i*,*k*)=−*∞* (first matrix entry)
- $L^{ijkl}(i,l')=G_{\!\text {A}}^{ijkl}(i,l')=LR^{ijkl}(i,l')=-\infty $ and $G_{\!\text {AB}}^{ijkl}(i,l')=0$ for all *l*^′^>*k* (first matrix row)
- $L^{ijkl}(j',k)=G_{\!\text {AB}}^{ijkl}(j',k)=LR^{ijkl}(j',k)=-\infty $ and $G_{\!\text {A}}^{ijkl}(j',k)=0$ for all *j*^′^>*i* (first matrix column)

By initializing the *LR* matrix with −*∞*, we keep matchings represented by *LR* and *L* distinct (because in this way, finite *LR* entries have to be derived via *G*_A_ or *G*_AB_ entries, which enforces a gap).

The final matrix *F* is initialized by *F*(*j*^′^,0)=*F*(0,*l*^′^)=0 for all *j*^′^,*l*^′^.

### ExpaRNA-P: suboptimal traceback & enumerating maximal EPMs

For enumerating only maximal EPMs during suboptimal traceback, we take special care that EPMs cannot be extended at the left or right end of gaps (*G*_A_ and *G*_AB_ matrices.) For strict EPMs this is decided independently of the other traced strict EPMs. It suffices to check whether the strict EPM can be extended into the gap matrices, i.e. whether a sequence or structure match is possible at the borders of the gap matrices.

However, the same does not work for relaxed EPMs, since while extending a relaxed EPM, the score might first decrease and then increase again (Figure 3). Therefore, we filter relaxed EPMs in two steps. First, we discard EPMs due to the same criterion as in the case of strict EPMs, checking for *exact* sequence or structure matches at the borders of the gap matrices. If an EPM cannot be discarded in this way, it is stored until all relaxed EPMs in the same D matrix are traced back. Only then, we compare the withheld relaxed EPMs of the same D matrix according to Def. 7.

Since we complete the whole traceback for a D matrix before tracing into its “enclosed” D matrices, we identify and remove all non-maximal relaxed EPMs in an early stage of the traceback.

To enumerate all maximal EPMs, we start such tracebacks only from entries *F*(*j*^′^,*l*^′^) that satisfy $A_{j^{\prime }+1} \neq B_{l^{\prime }+1}\phantom {\dot {i}\!}$. Due to Lemma 1, this condition is necessary and sufficient for strict EPMs.

#### **Lemma1**.

Let $\mathcal {P}=(\mathcal {M},\mathcal {S})$ be a maximal strict EPM of ${A}_{1..j^{\prime }}\phantom {\dot {i}\!}$ and ${B}_{1..l^{\prime }}\phantom {\dot {i}\!}$ with $j'\!\sim \!l'\in {\mathcal M}$.  is a maximal strict EPM of *A* and *B*, iff $A_{j^{\prime }+1} \neq B_{l^{\prime }+1}\phantom {\dot {i}\!}$.

#### *Proof*.

“ ⇒”: Let $A_{j^{\prime }+1}=B_{l^{\prime }+1}\phantom {\dot {i}\!}$. Then $\mathcal {P}':=(\mathcal { M}\cup \{j'+1\!\sim \!l'+1\},\mathcal {S})$ is a strict EPM with $\mathcal {P}\sqsubseteq \mathcal {P}'$; hence  is not maximal for *A* and *B* (i.e. among all strict EPMs of *A* and *B*). “ ⇐”: Let $A_{j^{\prime }+1} \neq B_{l^{\prime }+1}\phantom {\dot {i}\!}$.

Assume  is not maximal for *A* and *B*. Then, there is a strict EPM $\mathcal {P}'=(\mathcal {M}',\mathcal {S}')\neq \mathcal {P}$ with $\mathcal {P}\sqsubseteq \mathcal {P}'$ that is not a strict EPM of ${A}_{1..j^{\prime }}\phantom {\dot {i}\!}$ and ${B}_{1..l^{\prime }}\phantom {\dot {i}\!}$.

Consequently, to satisfy $\mathcal {M}\subset \mathcal {M}'$, there has to exist $ij\!\sim \!kl\in \mathcal {S}'$ with *i*≤*j*^′^<*j* and *k*≤*l*^′^<*l*. However in this case, while clearly the parent of *j*^′^ ∼ *l*^′^ in  is *ψ*, there is a parent of *j*^′^ ∼ *l*^′^ in $\mathcal {S}'$ different from *ψ* (i.e. either *ij* ∼ *kl* or some “smaller” base pair match). This contradicts $\mathcal {P}\sqsubseteq \mathcal {P}'$, because  and $\mathcal {P}'$ are not comparable by inclusion order (Def 6).

By the same argument, the forward direction holds for relaxed EPMs. Therefore, we enumerate all maximal relaxed EPMss by restricting the traceback in the same way. However, since the backward direction does not hold generally, this procedure can enumerate non-maximal relaxed EPMs. In practice, we observe this very rarely; consequently, while redundant relaxed EPMs could be removed explicitly, we let the chaining procedure handle those EPMs.

### ExpaRNA-P: Sparsification

ExpaRNA-P’s efficiency depends fundamentally on the sparsity of the DP matrices, which we leverage through fixed thresholds *θ*_1_,*θ*_2_, and *θ*_3_. Consequently, we compute all DP matrices in only *O*(*n*^2^) time and space. We compute matrices $L^{ijkl}, G_{\text {A}}^{ijkl}, G_{\text {AB}}^{ijkl}$, and *LR*^*ijkl*^ only for base pairs (*i*,*j*) and (*k*,*l*) that are significant (i.e. Pr[(*i*,*j*)|*A*]≥*θ*_1_ and Pr[(*k*,*l*)|*B*]≥*θ*_1_). Furthermore, we compute only relevant entries of these matrices.

This is best illustrated by the notion of candidates; each *j*^′^ is a *candidate of (i,j) in sequence A* if it is either a significant single-stranded position within (*i*,*j*), i.e. Pr[*j*^′^ ∈ loop(*i*,*j*)|*A*]≥*θ*_2_, or contained in a significant helix of (*i*,*j*), i.e. Pr[(*i*^′^,*j*^′^) ∈ loop(*i*,*j*)|*A*]≥*θ*_3_ for some *i*^′^. Analogously, we define *candidates l*^′^* of (k,l) in sequence B*. (For candidates *l*^′^ holds Pr[*l*^′^ ∈ loop(*k*,*l*)|*B*]≥*θ*_2_ or Pr[(*k*^′^,*l*^′^) ∈ loop(*k*,*l*)|*B*]≥*θ*_3_ for some *k*^′^).

#### **Theorem1**.

There are only *O*(*n*^2^) entries $L^{ijkl}(j',l'), G_{\text {A}}^{ijkl}(j',l')$, $G_{\text {AB}}^{ijkl}(j',l')$, and *LR*^*ijkl*^(*j*^′^,*l*^′^) such that *j*^′^ is a candidate of (*i*,*j*) and *l*^′^ is a candidate of (*k*,*l*). Consequently, ExpaRNA-P has quadratic time and space complexity.

***Proof sketch:*** By definition, only candidates *j*^′^ or *l*^′^ can be part of a significant EPM as defined in Def. 5; otherwise, we assign −*∞* to *L*^*ijkl*^(*j*^′^,*l*^′^) and *LR*^*ijkl*^(*j*^′^,*l*^′^). Furthermore, in the latter case, we neither store nor compute the values for $G_{\text {A}}^{ijkl}(j',l')$ and $G_{\text {AB}}^{ijkl}(j',l')$. Due to these considerations, in the matrices $L^{ijkl}, LR^{ijkl}, G_{\text {A}}^{ijkl}$, and $G_{\text {AB}}^{ijkl}$, we skip each complete row or column whose index is no candidate. Consequently, after computing a mapping from candidate sequence positions to matrix positions — independently for each sequence and for all significant base pairs, the sparsified algorithm operates on “contracted” matrices that contain only the candidate rows and columns. The first threshold *θ*_1_ reduces the number of base pairs to a constant number of base pairs per sequence position; in total, quadratically many base pairs pass the filter. The thresholds on joint probabilities guarantee that each sequence position is candidate of only constantly many base pairs. In consequence, each position is considered only a constant number of times during the entire computation; this directly results in quadratic time complexity. (Full proof in Additional file 1: Sec. 2)

### Chaining

Chaining selects a non-crossing and non-overlapping subset of EPMs. Our algorithm generalizes the chaining of ExpaRNA [30]. The chaining algorithm recursively fills the holes of all EPMs with other EPMs. For this purpose, it fills one *O*(*n*^2^) matrix for each hole and takes *O*(*Hn*^2^) time, where *H* is total number of holes with *H*≪*n*^2^. In contrast to ExpaRNA, there may exist more than one EPM ending at each sequence position pair, i.e. there is no one-to-one correspondence between EPMs and EPM’s end positions. This is why each matrix requires additional steps in the order of the number of input EPMs *E* in ExpaRNA-P’s chaining; the complexity of the generalized chaining algorithm is *O*(*H*·(*n*^2^+*E*)). Since in the most general case, when we enumerate all suboptimal EPMs up to a maximal difference to the optimal score, *E*∈*O*(*n*^2^) is not guaranteed, we implement in addition several ways to control the number of EPMs. For example, ExpaRNA-P allows setting an *ad hoc* limit on this number. Furthermore, we suggest a heuristic strategy: for each sequence position pair, keep only the best EPM ending there. Consequently, typical use cases of ExpaRNA-P maintain the chaining complexity of ExpaRNA, i.e. *O*(*Hn*^2^).

## Results and discussion

We implemented ExpaRNA-P and the chaining algorithm in C++. In particular, we implemented two versions of the traceback: the suboptimal traceback and a heuristic version that, for each match *i* ∼ *k*, considers only the optimal EPM ending at that match. Our tool supports two ways to control the EPM enumeration by the suboptimal traceback: either by defining the maximum score difference to the optimal score or the maximum number of EPMs.

In order to assess the performance of ExpaRNA-P, we designed the following pipeline: In a first step we compute the significant EPMs with ExpaRNA-P and use the chaining algorithm to extract from these EPMs an optimal non-overlapping and non-crossing subset. Then we compute a sequence structure alignment that includes all matches of the chained EPMs. For this purpose, we utilize the EPMs as anchor constraints for LocARNA. Consequently, LocARNA runs much faster, since each anchor reduces the alignment space. In correspondence with the analogous idea ExpLoc [30], which utilizes ExpaRNA anchors, we call our pipeline ExpLoc-P.

We performed all benchmarks over the pairwise alignment instances of the BRAliBase 2.1 benchmark set [44,45]. To measure the quality of the calculated alignment in comparison to the (for each instance) known reference alignment, BRAliBase 2.1 [44] provides the scoring tool compalignp. It computes the similarity between the two alignments as sum-of-pairs score (SPS). Identical alignments receive the SPS score 1; alignments without any correspondence, 0. In this way, we evaluated different variants of our method and later compare them to existing tools. At the same time, we opposed quality to runtime.

### Impact of EPM selection on the performance

We study five ExpLoc-P variants, where we generate anchor constraints respectively by

1. heuristic traceback with exact matches
2. heuristic traceback with inexact structure matches
3. suboptimal traceback with exact matches
4. suboptimal traceback with inexact structure matches
5. suboptimal traceback with inexact structure matches and the additional second filter step

In particular, we compare exact modes (1,3), which follow the strict EPM definition, and inexact modes (2,4,5), which allow mismatches at structure positions (relaxed EPMs.) The score parameters were selected ad-hoc without parameter learning; in particular, we set the cutoff probabilities to restrictive values *θ*_1_=*θ*_2_=*θ*_3_=0.01 to predict less false positives. Furthermore, we enumerated EPMs that have a score of at least 90 and fix the maximal number of traced EPMs in the suboptimal traceback to 100. The scoring – as defined in Eq. 1 and 2 was instantiated by setting the structure mismatch score *str_mm* to −10 for structure mismatches in inexact modes. Furthermore we set *α*_1_=1,*α*_2_=5 and *α*_3_=5 in order to favor structured regions. In addition to SPS and runtime, we computed the coverage for each benchmark instance – consisting of sequences *A* and *B*. For this purpose, we define *coverage* as the fraction of nucleotides that are matched by the best chain of EPMs $\mathcal {C} = \bigcup (\mathcal {M},\mathcal {S})$:

(3) $$ \text{coverage} = \frac{\sum_{(\mathcal{M},\mathcal{S}) \in \mathcal{C}} |\mathcal{M}|}{\min(A,B)}  $$

**Figure 6:**
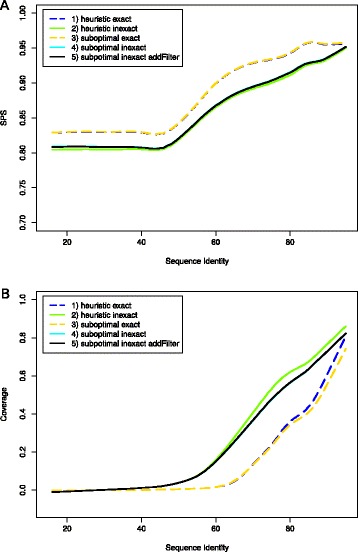
**Comparison of** **ExpLoc-P** **variants.** **(A)** Alignment quality (SPS) vs. sequence identity. **(B)** Coverage vs. sequence identity. Dependencies are visualized as LOWESS curves.

Figure 6A shows the alignment quality (SPS) versus the sequence identity; we visualized the dependency by estimating a LOWESS curve [46] for each series of benchmark evaluations. Overall, we observed that the difference between the suboptimal and heuristic traceback is not significant, solely for inexact modes, the suboptimal traceback leads to slightly better results. Furthermore, in inexact modes the additional second filter step did not change the quality significantly. Exact modes produced better alignments, however these modes generated much less anchor constraints for low sequence identity regions; in turn, the speedup decreases in these modes. This effect is visible in Figure 6B, which plots the estimated coverage vs. the sequence identity. The exact modes predict EPMs only for sequence identity values above 60%. For the inexact modes, we obtained much higher coverage; notably, we predicted many more relaxed EPMs than strict EPMs for the sequence identity interval from 40-60%.

In Table 1, we report total runtimes and average SPS scores of different ExpLoc-P variants over the entire benchmark set. Furthermore, we provide single timings for preprocessing (first value in brackets), computing and chaining the EPMs (second value), and subsequent LocARNA alignments (third value). The differences in coverage directly impact the runtimes of the different variants, but not as pronounced, since – like one would expect for many real world applications – the benchmark set contains many high identity sequences. Consequently, relaxed EPMs significantly reduced the runtime for instances with sequence identity between 50-80% (Additional file 1: Figure S3A). Furthermore, the heuristic traceback was slightly faster than the suboptimal one for long RNA sequences (Additional file 1: Figure S3B), while suboptimal traceback could not significantly improve the alignment quality in this setting. Consequently, for this specific benchmark, the two variants with heuristic traceback turned out to provide the best balance of quality and speedup.

**Table 1:** **Comparison of** **ExpLoc-P** **variants**

**ExpLoc-P** ** variant**	**1**	**2**	**3**	**4**	**5**
Total time	3.5 h	3.0 h	3.7 h	3.1 h	3.1 h
	(0.6 h + 0.4 h + 2.6 h)	(0.6 h + 0.5 h + 1.9 h)	(0.6 h + 0.4 h + 2.7 h)	(0.6 h + 0.5 h + 2.0 h)	(0.6 h + 0.5 h + 2.0 h)
Total SPS	0.86	0.84	0.86	0.84	0.84

### Comparison to other tools

We benchmarked three existing approaches: LocARNA, ExpLoc [30], and RAF [29]. LocARNA without anchors serves as base line approach; in contrast to ExpLoc-P, ExpLoc identifies EPMs in a single predicted structure for each RNA (using ExpaRNA); and RAF is currently the fastest Sankoff-style alignment approach due to its heuristic filtering based on sequence alignments. We compared these approaches to ExpLoc-P variants 1 and 2, which performed best in the previous section.

**Figure 7:**
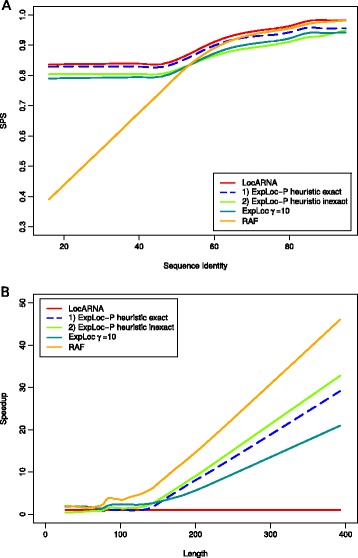
**Comparison with sequence-structure alignment methods.** **(A)** Comparison of alignment quality vs. sequence identity. **(B)** Comparison of speedup over LocARNA vs. length (LOWESS).

Table 2 summarizes the results; we report the speedup over LocARNA, total runtime, and average alignment quality (SPS) across the entire benchmark set (Opteron 2356, 2.3 GHz, single thread). Figure 7A shows the behavior of the compalignp score dependent on the sequence identity. LocARNA aligned with the best quality at the expense of the highest computation time. The best alignment quality that has been obtained with ExpLoc in [30] has been achieved with parameter minsize=10. Even this quality is significantly lower than the one for the two variants of ExpLoc-P (0.81 vs. 0.84 and 0.86). Moreover, the overall speedup for this setting is not much higher than the speedups for ExpLoc-P. Although RAF achieved the best speedup of 14.4, the quality drops tremendously for sequence similarities below 50%.

**Table 2:** **Comparison of RNA alignment methods**

	**LocARNA**	**ExpLoc-P**	**ExpLoc-P**	**ExpLoc**	**RAF**
		**(variant 1)**	**(variant 2)**	***γ*** **=10**	
Speedup	1	3.9	4.6	5.0	14.4
Runtime	13.8 h	3.5 h	3.0 h	2.8 h	1.0 h
SPS	0.87	0.86	0.84	0.81	0.86

The quality drop of RAF alignments at low sequence identities is strongly reminiscent of pure sequence alignment methods. Thus, we conjecture that the specific use of sequence-based heuristics by RAF, while guaranteeing sequence alignment like run-time behavior, compromises RAF’s use for ‘hard’ RNA alignment instances that require structure-based alignment methods.

Furthermore, we investigated the dependency of the lengths of the input sequences on the speedup (see Figure 7B). As expected, the speedup increased for longer input sequences. For RNA sequences longer than 150 bases, we obtained a significantly better speedup with both variants of ExpLoc-P compared to ExpLoc. Moreover, the speedup difference increases with the lengths of the input sequences (Additional file 1: Figure S4 provides a detailed comparison of ExpLoc-P variants 1 and 2). For the longest input sequences, ExpLoc-P achieved respective speedups of 32 and 35 for variants 1 and 2, and RAF of almost 50.

To summarize, ExpLoc-P provided the best trade-off between alignment quality and speedup in this setting; robustly, it maintained high alignment quality over the entire range of sequence identities; finally, it proofed to be particularly suited for long instances.

## Conclusion

We have introduced the algorithm ExpaRNA-P that very efficiently identifies exact pattern matches in RNAs by matching and folding them simultaneously. The method is a major achievement over previous approaches (including the “predecessor” ExpaRNA) that – without being more efficient – are much less flexible, since they require a priori known or unreliably predicted structure.

Due to its novel ensemble-based sparsification, the algorithm ExpaRNA-P has only a very low (quadratic) time and space complexity, equalling sequence alignment. This sparsification technique is particularly relevant, since similar techniques can likely be applied to other RNA analysis methods.

We have developed two major variants of this method; one requires strict matches in all positions of an EPM (strict EPMs), the other relaxes this (therefore, relaxed EPMs) to allow mismatches at structural positions. The latter supports compensatory mutations, which are highly relevant in RNA structure analysis in general.

Our benchmarks study EPMs as anchor constraints to speed up RNA structure alignments (in the form of simultaneous alignment and folding by LocARNA). EPMs from structure ensembles have turned out to be substantially more reliable than EPMs from fixed structures. At comparable speed ups, this results in increased quality. Most importantly, the novel approach keeps up the alignment quality even for sequences of low identity, which is ultimately decisive for structure alignment. In striking contrast, the alignment quality of the similarly fast alignment tool RAF breaks down – very much like pure sequence alignment.

We have implemented rigorous suboptimal traceback, which provides extensive control of the set of enumerated EPMs. For example, this level of control is required in the analysis of structural variants common to the RNAs. In addition, we have developed a heuristic traceback, which performs almost indistinguishable in our benchmark. Being much faster than the rigorous method, it offers the best speed-quality balance in such settings.

Finally, we conjecture that EPM-based anchor constraints can be combined advantageously with other RNA alignment tools such as RAF. While for LocARNA the constraints yield a considerable speedup, the combination with RAF has the potential to improve RAF’s poor alignment quality for low sequence similarity.

## Additional file

Additional file 1
**Supporting Information.** Supplementary document containing details about precomputing joint loop probabilities (including a complexity analysis), the proof of ExpaRNA-P’s complexity and supplementary results.
