# Supplementary material for: ExpaRNA-P: simultaneous exact pattern matching and folding of RNAs
Source: BMC Bioinformatics. 2014 Dec 31;15(1):404. doi: 10.1186/s12859-014-0404-0 (PMC4302096; doi:10.1186/s12859-014-0404-0)
Supplement: Additional file 1 — Supporting Information. Supplementary document containing details about precomputing joint loop probabilities (including a complexity analysis), the proof of ExpaRNA-P’s complexity and supplementary results. [file 12859_2014_404_MOESM1_ESM.pdf]

Additional file 1: Supporting Information

ExpaRNA-P: Simultaneous Exact Pattern Matching and Folding of RNAs

1 Precomputing joint loop probabilities

1.1 Computation of unpaired probabilities in loops

We present the computation for sequence A (the case of B is analogous.) We compute base pair probabilities $\Pr[(i,j)|A]$ by the McCaskill algorithm [1]. Furthermore, we extend this algorithm to compute the probabilities $\Pr[k \in \text{loop}(i,j)|A]$ and $\Pr[(i',j') \in \text{loop}(i,j)|A]$. For this purpose, we utilize the matrices Q_{ij}, Q^b_{ij}, Q^m_{ij}, and Q^{m1}_{ij} as defined by [1]. To recapitulate this briefly: for $1 \leq i \leq j \leq |S|$, the entries of these matrices equal the respective sums over the Boltzmann weights of the following sets of structures of $A_{i..j}$

- Q_{ij}: all structures of $A_{i..j}$
- Q^b_{ij}: all structures S of $A_{i..j}$ with $(i,j) \in S$
- Q^m_{ij}: all non-empty structures of $A_{i..j}$ scored as part of a multiloop
- Q^{m1}_{ij}: all structures S of $A_{i..j}$, scored as part of a multiple loop, such that for some k holds $(i,k) \in S$ and for all $(i',j') \in S$ holds $i \leq i' < j' \leq k$.

Intuitively, Q^{m1}_{ij} counts the Boltzmann weights of all structures that are part of a multiloop and have exactly one outermost base pair, starting at position i. Extending the original set of matrices, we compute the additional matrix $Q^{m2}_{ij} = \sum_{i<k<j-1} Q^m_{ik} Q^{m1}_{k+1\,j}$. It represents parts of a multiloop with at least two outermost base pairs. Note that this matrix is computed in $O(n^3)$ time without adding to the complexity of McCaskill's algorithm. Given those matrices, we compute $\Pr[k \in \text{loop}(i,j)|A]$ as

$$\Pr[k \in \text{loop}(i,j)|A] = \Pr[(i,j)|A] \frac{H + I + M}{Q^b_{ij}},$$

where

$$H = \exp(-\beta F_1(i,j))$$

$$I = \sum_{\substack{i',j' \\ i<k<i'<j'<j}} \exp(-\beta F_2(i,j,i',j')) Q^b_{i'j'} + \sum_{\substack{i',j' \\ i<i'<j'<k<j}} \exp(-\beta F_2(i,j,i',j')) Q^b_{i'j'}$$

$$M = Q^{m2}_{k+1 j-1} \exp(-\beta(a + (k-i)c)) + Q^{m2}_{i+1 k-1} \exp(-\beta(a + (j-k)c)) + Q^m_{i+1 k-1} Q^m_{k+1 j-1} \exp(-\beta(a + c))$$

Please compare the formulas to the visualization in App. Fig. 1. H, I, and M are conditional partition functions, conditioned by $k \in \text{loop}(i,j)$, in the respective cases where k is contained in a hairpin, interior loop, and multiloop. The constants a, c, k, and T, as well as the energy functions F_1 (hairpin loop) and F_2 (interior loop) are defined as in McCaskill [1], where $\beta := (kT)^{-1}$. To compute M, we distinguish three cases: k is either in the leftmost, the rightmost, or any other unpaired region of the loop. Note that in the computation of M, one needs to ensure that M considers only multiloops, which must have at least two inner base pairs. Only the matrix Q^{m2} enables performing this in constant time.

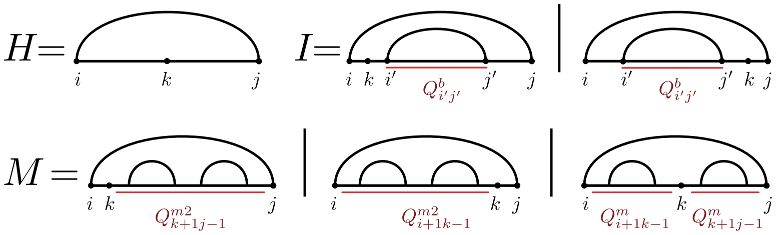

Additional file 1: Figure S1 Unpaired probability recursions. Recursions of the partition functions for computing the probability that a position k occurs inside a hairpin loop (H), interior loop (I), or multiloop (M) closed by a base pair (i, j).

1.2 Computation of base pair probabilities in loops

The computation of $\Pr[(i', j') \in \text{loop}(i, j)|A]$ is similar to the computation of $\Pr[k \in \text{loop}(i, j)|A]$. More precisely we compute $\Pr[(i', j') \in \text{loop}(i, j)|A] \geq \theta_3$ for all (i, j), (i', j') with $\Pr[(i, j)|A] \geq \theta_1$ and $\Pr[(i', j')|A] \geq \theta_1$ as

$$\Pr[(i', j') \in \text{loop}(i, j)|A] = \Pr[(i, j)|A] \frac{I' + M'}{Q^b_{ij}}, \text{ where}$$

$$\begin{aligned} I' &= \exp(-\beta F_2(i, j, i', j')) Q^b_{i'j'} \\ M' &= \exp(-\beta(a + (i' - i - 1)c) Q^b_{i'j'} Q^m_{j'+1j-1} \\ &\quad + Q^m_{i+1i'-1} Q^b_{i'j'} \exp(-\beta(a + (j - j' - 1)c) \\ &\quad + Q^m_{i+1i'-1} Q^b_{i'j'} Q^m_{j'+1j-1} \exp(-\beta(a)) \end{aligned}$$

As visualized in App. Fig. 2, (i', j') can either be contained in an internal loop (case I') or a multiloop (case M'). In the multiloop case, we distinguish the three subcases where (i', j') is the leftmost, the rightmost or some other inner base pair of the loop.

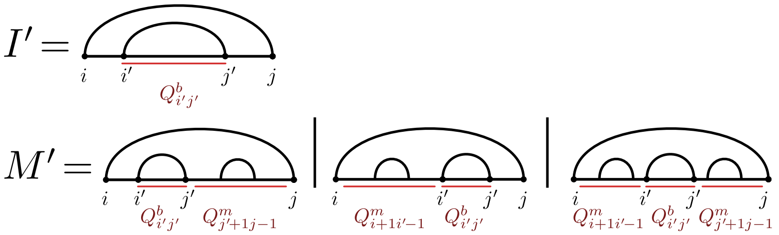

Additional file 1: Figure S2 Base pair probability recursions. Computation of the probability that a base pair (i', j') occurs inside an interior loop (I') or multiloop (M') closed by a base pair (i, j).

1.3 Complexity Analysis

We compute $\Pr[(i, j)|A]$ for all $1 \leq i < j \leq n$, $\Pr[k \in \text{loop}(i, j)|A]$ for all (i, j) with $\Pr[(i, j)|A] \geq \theta_1$ and $\Pr[(i', j') \in \text{loop}(i, j)|A]$ for all (i, j), (i', j) with $\Pr[(i, j)|A] \geq \theta_1$, $\Pr[(i', j')|A] \geq \theta_1$.

Since for some fixed j, each structure of A can have at most one base pair ending at j, there exist at most $\frac{1}{\theta_1}$ base pairs (i, j) for each j with $\Pr[(i, j)|A] \geq \theta_1$. Hence,

in total there exist at most $n\frac{1}{\theta_1} \in O(n)$ base pairs (i,j) with $\Pr[(i,j)|A] \geq \theta_1$, and consequently we need to compute only $O(n^2)$ values. The only value that requires more than constant time to compute is the computation of I. In the same way as in the McCaskill algorithm this computation can be reduced to linear time by restricting the loop length. Hence, all $O(n^2)$ values together can be computed in $O(n^3)$ time.

2 Complexity Analysis of ExpaRNA-P

Lemma 1 *For a fixed j', there are only $O(1)$ base pairs (i,j), such that j' is a candidate of (i,j) (and analogously for l' and (k,l) in sequence B).*

Proof We fix some j' and denote by $p_{j'}(i,j)$ the probability that a structure of A contains the base pair (i,j) and j' occurs as an unpaired base or right end of a base pair in the loop closed by the base pair (i,j): $p_{j'}(i,j) := \Pr[j' \in \text{loop}(i,j)|A] + \sum_{i<i'<j'} \Pr[(i',j') \in \text{loop}(i,j)|A]$. If j' is a candidate, it follows $p_{j'}(i,j) \geq \theta^* := \min\{\theta_2, \theta_3\}$, since then either $\Pr[j' \in \text{loop}(i,j)|A] \geq \theta_2$ or $\Pr[(i',j') \in \text{loop}(i,j)|A] \geq \theta_3$ for some i'. Note that for different (i,j) the events of probabilities $p_{j'}(i,j)$ are disjoint, since in any structure j' can occur in just one loop. Therefore $\sum_{i,j} p_{j'}(i,j) \leq 1$. Hence there are at most $\frac{1}{\theta^*} \in O(1)$ base pairs (i,j) for which $p_{j'}(i,j) \geq \theta^*$ and only for those j' can be a candidate. □

Note that for this lemma it is crucial to consider the probabilities within the single loops and not only general base pair and unpaired probabilities. Considering these probabilities is the key insight of this new way of sparsification.

Theorem 1 *There are only $O(n^2)$ entries $L^{ijkl}(j',l')$, $G_A^{ijkl}(j',l')$, $G_{AB}^{ijkl}(j',l')$, and $LR^{ijkl}(j',l')$ such that j' is a candidate of (i,j) and l' is a candidate of (k,l).*

Proof Due to Lem. 1 there are $O(n)$ many combinations i, j, j'. Analogously there are $O(n)$ combinations k, l, l' and therefore $O(n^2)$ combinations i, j, k, l, j', l' satisfying the conditions. □

Corollary 1 *The time and space complexity of computing all entries $L^{ijkl}(j',l')$, $G_A^{ijkl}(j',l')$, $G_{AB}^{ijkl}(j',l')$, and $LR^{ijkl}(j',l')$, D and F is $O(n^2)$.*

Proof We show that D has $O(n^2)$ entries. Clearly, the number of all other matrix entries is quadratically bounded due to Theorem 1. The argument for D has been given before in the context of LocARNA [2]; also compare the proof of Lemma 1. For sequence A (analogously, for B) and each position j, there exist only $O(1)$ base pairs (i,j), where $\Pr[(i,j)|A] \geq \theta_1$. For fixed j, the probabilities of base pairs (i,j) sum up to at most one; since each probability is at least θ_1, there are at most $1/\theta_1$ such base pairs.

For the same reason, each matrix entry is computed in constant time: whenever we iterate over base pairs $(i,j) \in P$ and $(k,l) \in Q$, we require $\Pr[(i,j)|A] \geq \theta_1$ and $\Pr[(k,l)|B] \geq \theta_1$. Hence, for fixed j and l, these iterations are constantly bounded. □

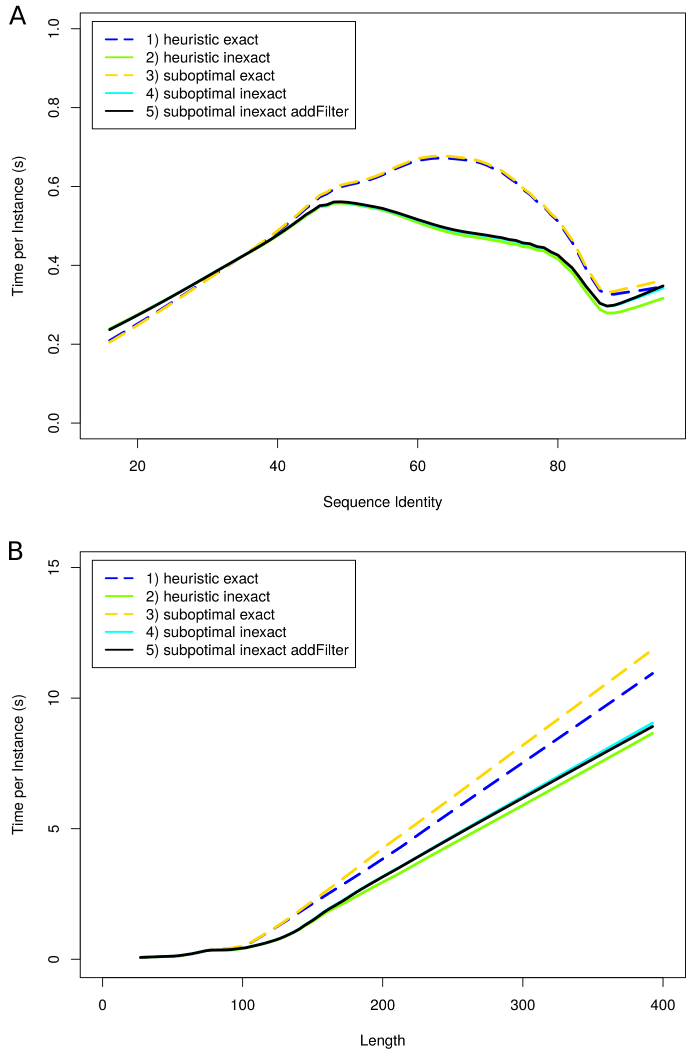

Additional file 1: Figure S3 Comparison of ExpLoc-P variants. **(A)** Runtime vs. sequence identity **(B)** Runtime vs. length. The length of an instance is defined as the average length of its two sequences.

3 Supplementary Results

App. Figs. 3A and 3B show the dependency of run-time on sequence identity and length over the benchmark set. Furthermore, App. Fig. 4 compares the speedup distributions of two ExpLoc-P variants as boxplot. The benchmark instances are classified according to their sequence length in the intervals (0,50], (50,100], (100,150], (150,200], (200,250], (250,300], (300,350], and (350,400]. For sequences of lengths greater than 150 we achieve substantial speedup. The inexact mode is also superior to the exact mode for these input sequences. For the longest input sequences, we even obtain respective speedups of 32 and 35 for the exact and inexact mode. These results show that utilizing anchor constraints to speed up sequence-structure alignments pays off especially if RNAs get longer. Note that while our benchmarks are rather biased to short instances, they clearly show the effect.

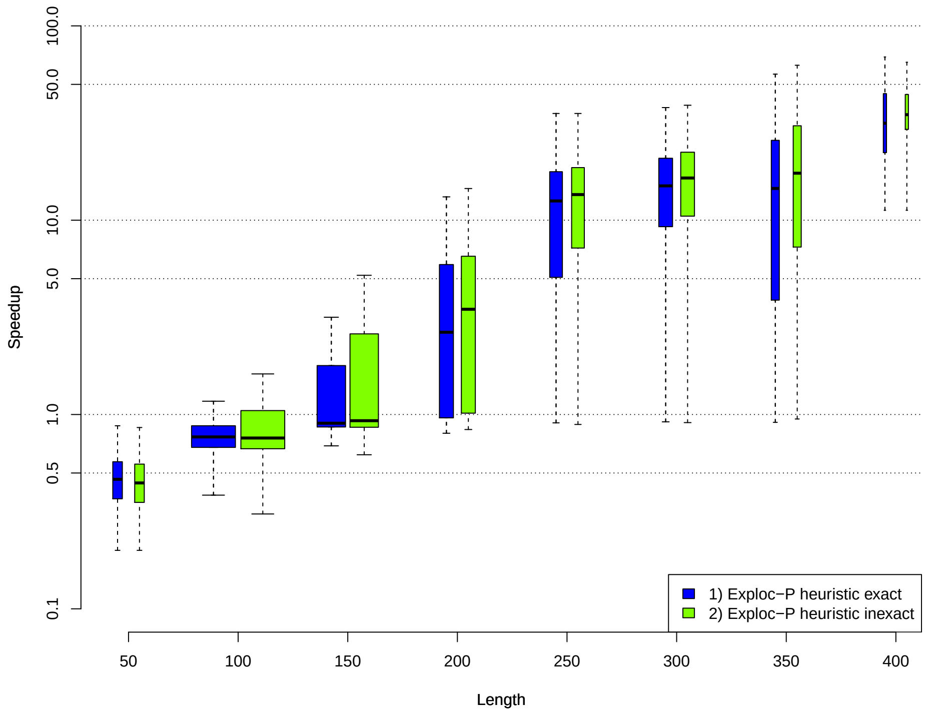

Additional file 1: Figure S4 Boxplot for the speedup over LocARNA vs. sequence lengths for ExpLoc-P variants. The speedup is measured relative to the speed of LocARNA.

Author details

References

1. McCaskill, J.S.: The equilibrium partition function and base pair binding probabilities for RNA secondary structure. Biopolymers **29**(6-7), 1105–19 (1990)
2. Will, S., Reiche, K., Hofacker, I.L., Stadler, P.F., Backofen, R.: Inferring non-coding RNA families and classes by means of genome-scale structure-based clustering. PLOS Computational Biology **3**(4), 65 (2007)
